# Genetically Engineered Bacterial Biohybrid Microswimmers for Sensing Applications

**DOI:** 10.3390/s20010180

**Published:** 2019-12-28

**Authors:** Zhiyong Sun, Philipp F. Popp, Christoph Loderer, Ainhoa Revilla-Guarinos

**Affiliations:** 1 Department of Molecular Biotechnology, Institute für Mikrobiologie, Technische Universität Dresden, 01217 Dresden, Germany; 2 Department of General Microbiology, Institute für Mikrobiologie, Technische Universität Dresden, 01217 Dresden, Germany

**Keywords:** bacterial biohybrid microswimmers, sensing, two-component system, fluorescence readout, GRAS, *Bacillus subtilis*

## Abstract

Bacterial biohybrid microswimmers aim at exploiting the inherent motion capabilities of bacteria (carriers) to transport objects (cargoes) at the microscale. One of the most desired properties of microswimmers is their ability to communicate with their immediate environment by processing the information and producing a useful response. Indeed, bacteria are naturally equipped with such communication skills. Hereby, two-component systems (TCSs) represent the key signal transducing machinery and enable bacteria to sense and respond to a variety of stimuli. We engineered a natural microswimmer based on the Gram-positive model bacterium *Bacillus subtilis* for the development of biohybrids with sensing abilities. *B. subtilis* naturally adhered to silica particles, giving rise to different motile biohybrids systems with variable ratios of carrier(s)-to-cargo(es). Genetically engineered TCS pathways allowed us to couple the binding to the inert particles with signaling the presence of antibiotics in their surroundings. Activation of the antibiotic-induced TCSs resulted in fluorescent bacterial carriers as a response readout. We demonstrate that the genetically engineered TCS-mediated signaling capabilities of *B. subtilis* allow for the custom design of bacterial hybrid microswimmers able to sense and signal the presence of target molecules in the environment. The generally recognized as safe (GRAS) status of *B. subtilis* makes it a promising candidate for human-related applications of these novel biohybrids.

## 1. Introduction

**Figure 1:**
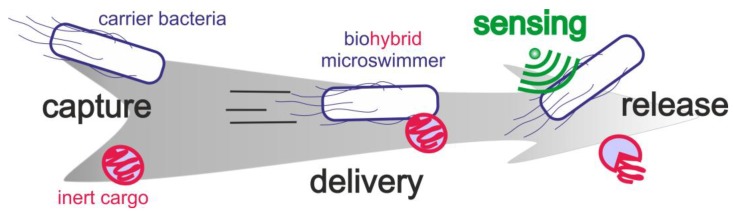
Bacterial biohybrid microswimmers development in a nutshell—capture, delivery, sensing, and release.

Bacteria have been intensively studied for centuries. Nevertheless, today, a new field of research highlights a previously un-envisioned application of these prokaryotic microorganisms that is currently gathering momentum—their potential for the development of microswimmers. Bacterial biohybrid microswimmers usually consist of one (or many) living bacteria and one (or many) abiotic particles [1]. Depending on the bacteria-to-particle ratio, the hybrid microswimmer can be classified as a Bacteria Bot (1 bacterium:1 cargo) (examples in references [2,3,4]), a Multi Bot (1 bacterium:>1 cargoes) (examples in references [5,6]), or a Bacterial Microsystem (>1 bacteria:1 cargo) (examples in references [7,8]). In these microswimmers, the bacteria usually act as carriers that provide the driving force required to move the inert cargoes from an initial point A to a final point B, i.e., bacterial metabolism hereby provides the chemical energy required for this microscale mechanical transport process. Three critical stages can be identified during the creation of these self-propelled bacterial biohybrid microswimmers—the capture, the delivery, and the release of the cargo (Figure 1). It is important to emphasize that one desired property of these microswimmers is their ability to perceive and react to environmental cues in an autonomous way (Figure 1). All these critical challenges on the developed biohybrid microswimmers are strongly determined by the bacterial characteristics.

The composition of the bacterial surface determines its attachment properties [9,10] and has a direct influence on the initial cargo capture and, potentially, the final cargo release. Gram-positive and Gram-negative bacteria are encased in a multilayered cell envelope with different architectures. In Gram-positive bacteria, the cytoplasmic membrane is surrounded by a thick cell wall of peptidoglycan; in Gram-negative bacteria, the cytoplasmic membrane is surrounded by a thin layer of peptidoglycan and an additional outer membrane called the lipopolysaccharide layer [11]. In both cases, the bacterial surface net charge is predominantly negative, and its electrostatic properties are influenced by the ionization of the functional groups from the macromolecules present in the cell walls and membranes [12]. Surface differences like the length of lipopolysaccharide molecules or the presence/absence of extracellular polymeric substances result in different adhesions of bacteria to inorganic surfaces [13]. Consequently, one approach to improve the attachment of bacteria to their cargo consists of modifying the bacterial surface composition. For example, Gram-negative *Escherichia coli* cells were modified to express biotin on their surface, thereby allowing the binding to streptavidin-functionalized microparticles [14]. Another approach is to use the natural electronegativity of the cells to bind positively charge molecules. For example, Gram-positive *B. subtilis* cells were attached to amino-functionalized zeolite L crystals [3].

The delivery of the cargo depends on the type of bacterial locomotion. Three different active movements of bacteria have been used for the development of bacterial biohybrid microswimmers, as reviewed in reference [1]—swimming, swarming, and gliding motilities. Swimming and swarming both depend on the use of flagella [15,16]. Differential flagellation patterns result in different possibilities for movement trajectories like the run-and-tumble swimming of *E. coli* [17] or the forward, reverse, and turning by buckling of *Vibrio alginolyticus* [18]. These swimming properties are exploited for the development of biohybrid microswimmers [19]. Furthermore, besides the self-actuated biohybrid microswimmers with uncontrolled motion (due to the action of individual cells in the absence of stimuli [20]), the bacterial taxis mediated by specific receptors and signal transduction pathways can be used to steer the directionality of the cargo transport toward or away from specific stimuli [5,21].

**Figure 2:**
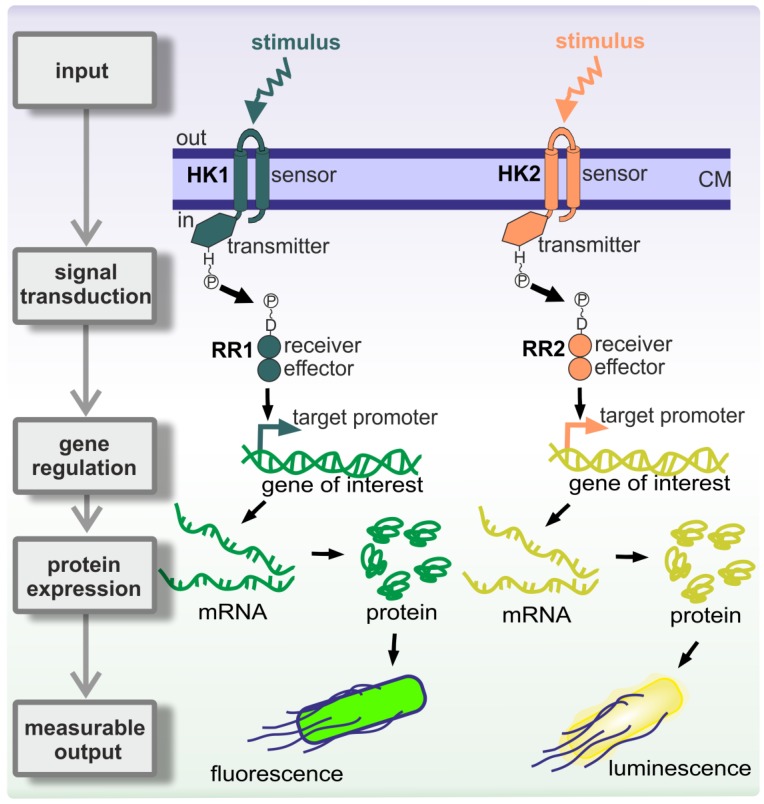
Two-component system (TCS)-based signal transduction in bacteria connects specific inputs with measurable output responses. Schematic showing two TCSs sensing two different external inputs (stimulus), leading to the activation of their corresponding target promoters and concomitant gene expression. This results in expression of green fluorescent protein in the case of TCS1 or luminescence in the case of TCS2. In both cases, the response can be experimentally measured. See main text for details on TCS signal transduction mechanistic. HK, histidine kinase; RR, response regulator; CM, cell membrane.

As previously mentioned, a desired property of microswimmers is the possibility of responding to environmental conditions while autonomously moving. At the single cell level, communication can be understood as the ability to perceive information from the environment, processing of this information, and emitting an appropriate response to it (usually to improve survival chances) [22]. In bacteria, besides other molecular strategies [23], two-component system (TCS)-mediated signal transduction pathways connect specific stimulus (inputs) to the adequate cellular responses (outputs) [24] (Figure 2).

Two-component systems are usually composed of a membrane-anchored sensor histidine kinase (HK) and a cytoplasmic response regulator (RR) (Figure 2) [25]. The HK perceives a specific stimulus (=input; e.g., an environmental cue) and auto phosphorylates at a histidine residue [26]. The high-energy phosphate group is subsequently transferred to an aspartyl residue on the RR, resulting in its activation [24]. RRs usually act as transcriptional activators/repressors and its activation results in the up-/down-regulation of target gene expression, whose products are required for the response (=output) to the specific input that activated the signal transduction pathway [27]. TCS have very interesting features for the development of genetic tools for applied purposes such as whole-cell biosensors [28]. For that purpose, the target promoters of the RRs are placed upstream of reporter genes, such as those encoding fluorescent proteins or luciferase systems (Figure 2). Regulated promoters usually show a dose-dependent response [29]; that is, the fluorescent or luminescent output is a function of the input strength.

Another important aspect of biohybrids is their biological safety. For example, despite the claims of using bacterial biohybrid microswimmers for human health applications, such as drug delivery (reviewed in reference [30]), the references to the investigation of probiotic bacteria [31] or microorganisms with granted Qualified Presumption of Safety (QPS, from the European Food Safety Authority (EFSA)) [32] or Generally Recognized as Safe (GRAS, from the US Food and Drug Administration (FDA)) status as biohybrid carriers, are scarce [3,33,34]. Interestingly, most probiotic bacteria (e.g., Lactobacilli and Bifidobacteria) [31] and bacteria with QPS status [35] are Gram-positives. However, literature review shows that Gram-negative bacteria such as *E. coli*, *Serratia marcescens*, or *Salmonella typhimurium* have been widely used for the development of bacterial biohybrids, and Gram-positive bacteria have been less investigated (reviewed in [1]).

In this study we developed the potential of the Gram-positive, QPS/GRAS status, model bacterium *B. subtilis* for the creation of bacterial hybrid microswimmers with sensing and signaling capabilities. Molecular microbiology techniques allowed the creation of whole-cell biosensors based on two TCS pathways of *B. subtilis*: LiaRS [36] and BceRS [37]. Our results show that *B. subtilis* naturally adheres to silica particles, thereby giving rise to different variations of hybrid microswimmers. Carriers with one bacterium or with cell chains moved in different ways. As a proof of concept for the sensing capabilities, we used the activation of the TCS pathways LiaRS and BceRS by the inducing antibiotic bacitracin: two genetically different populations of biohybrids within the same sample communicated the presence of the antibiotic by different fluorescent profiles. Our approach provides the molecular tools for the development of the ideal biohybrids with autonomous sensing, computation and actuation properties. Biohybrids that can perceive multiple environmental stimuli simultaneously and are only limited by the number of their natural signal transduction pathways.

## 2. Materials and Methods

### 2.1. Bacterial Strains, General Growth Conditions, and Swarming Growth Conditions

The bacterial strains used in this study are listed in Table 1. *B. subtilis* strains were grown in Luria-Bertani medium (LB-Medium (Luria/Miller), Carl Roth, Karlsruhe, Germany) at 37 °C with aeration. Agar-Agar (Kobe I, Carl Roth, Karlsruhe, Germany) at 1.5% (*w*/*v*) was added to prepare the corresponding solid media. The strains were stored at −80 °C in their corresponding growth media containing 50% (*v*/*v*) glycerol (Carl Roth, Karlsruhe, Germany). Chloramphenicol 5 μg mL^−1^, erythromycin 1 µg mL^−1^, and lincomycin 25 µg mL^−1^ (all three purchased from Sigma-Aldrich, Merck KGaA, Darmstadt, Germany) were added to *B. subtilis* strains for selection when required.

The assay for the induction of swarming movement of *B. subtilis* in soft-agar media was adapted from reference [40]. Briefly, 25 mL of LB−0.3% (soft) agar plates were prepared, with antibiotics for selection when required, in 100 mm diameter Petri dishes (Carl Roth, Karlsruhe, Germany). The melted agar medium was allowed to cure overnight at room temperature with the lids on. The soft-agar plates were inoculated immediately after the drying period was over. Overnight cultures of the strains under study were prepared with antibiotic selection when required. Day cultures (10 mL) were inoculated 1:200 with overnight cultures and incubated at 37 °C (220 rpm) without antibiotic selection until an OD_600_ of around 0.5–1 was reached. Then, three µL of the day cultures cell suspensions were used to inoculate the cured soft-agar swarm plates. The plates were allowed to dry before moving and were incubated upside down overnight at 37 °C inside a plastic bag. Bacteria–particle binding assays were performed after this incubation period.

### 2.2. Dose Response Induction of Fluorescence Proteins

The characterization of the dose response induction of green fluorescence protein coupled to the activation of the LiaRS two-component system signaling pathway was performed as previously described in reference [39] and modified as follows. Overnight cultures of strain P*_liaI_*-sfGFP (TMB3909, Table 1) prepared in LB with chloramphenicol selection were used to inoculate day cultures in LB at a 1:50 dilution ratio without further antibiotic selection. At an OD_600_ around 0.8–1, the cultures were split in five aliquots—one of them was left as uninduced control, and the other four were induced with bacitracin (Sigma-Aldrich, Merck KGaA, Darmstadt, Germany) at final concentrations 0.3, 1, 10, and 30 µg mL^−1^. After a further incubation for 75 min to allow fluorescent protein production, two mL of each culture were harvested by centrifugation and were washed once with one mL phosphate buffered saline (PBS, Sigma-Aldrich, Merck KGaA, Darmstadt, Germany). The cells were finally resuspended in 1 mL PBS, and 200 μL of each resuspension were filled into each well of a 96-well plate (black, clear bottom; Greiner Bio-One, Frickenhausen, Germany). Quantification of the sfGFP endpoint fluorescence was performed by reading the plates from the top without lid using the Synergy^™^ NeoalphaB plate reader (BioTek, Winooski, VT, USA) with monochromators for illumination. Excitation and emission wavelengths were fixed at 481 nm and 511 nm, respectively; the gain was fixed to 100; a xenon flash light source with high energy lamp was used; the read speed was set to normal, 100 msec delay. For normalization, fluorescence values were divided by the amount of cells as determined by OD_600_. Mean and standard deviation were determined for four biological replicas, each of them with a technical duplicate.

### 2.3. Bacteria-Particle Binding Assays

Bacteria on the leading edge of the swarming colonies were suspended in Milli-Q^®^ (Merck KGaA, Darmstadt, Germany) water. Different dilutions of swarming bacteria were mixed with sonicated water suspensions of the abiotic particles (total final volume of 50 µL), in order to reach an approximate ratio of 50% bacteria −50% particles as estimated by microscopical observation of the preparations. The suspensions were incubated at 37 °C (550 rpm) for at least 30 min to promote bacteria-particle interactions prior to the microscopic observation of the biohybrid microswimmers. When fluorescent protein expression was required, the expression of the fluorescent proteins under the control of the bacitracin inducible promoters P*_liaI_* and P*_bceA_* was achieved by addition of bacitracin at a final concentration of 18 µg mL^−1^ or 30 µg mL^−1^ (see figure legends), and the incubation was carried out for one hour prior to the microscopic observation.

### 2.4. Optical Microscopy

For the microscopic observations, 5 µL of the bacteria–particles preparations were deposited on sterile four-chamber glass bottom 35 mm dish with 20 mm bottom well plates, from In Vitro Scientific (ref. D35C4-20-1.5-N). The cells were observed with an Axio Observer.Z1/7 ZEISS fluorescence microscope with Plan-Apochromat 100×/1.40 Oil DIC M27 objective, and ZEN 2.3 pro software (ZEISS, Jena, Germany). Pictures were made with an AxioCam 702 m left, ZEISS camera (ZEISS, Jena, Germany). Microscopy pictures and videos were analyzed using the tools implemented in the MicrobeJ (ImageJ 1.52 i) and MTrackJ (for calculation of biohybrids velocities) software [41,42,43].

Phase contrast microscopy pictures were made with light source TL LED at 7.52 Volt, exposure time of 25 ms, and depth of focus of 0.85 µm. Phase contrast videos were recorded at 25 frames per second. When fluorescent bacteria were visualized, up to three channels were used for bright field, green fluorescence and red fluorescence (if required). Bright field contrast method parameters were as follows: light source, TL LED, 3.00 Volt; exposure time, 4.6 s; depth of focus, 0.85 µm. The parameters for green fluorescence recording were as follows: contrast method, fluorescence; light source, LED-Module 475 nm at 100% intensity; illumination wavelength, 450–488 nm; excitation, 488 nm; emission, 509 nm; exposure time, 800 ms; depth of focus, 0.79 µm. The parameters for red fluorescence recording were as follows: contrast method, fluorescence; light source, LED-Module 567 nm at 100% intensity; illumination wavelength, 577–604 nm; excitation, 587 nm; emission, 610 nm; exposure time, 800 ms; depth of focus, 0.94 µm. For video recording of the green fluorescent microswimmers movement, we performed 10-frame (71.7 s) time series with two channels for green fluorescence and bright field.

### 2.5. Particles: Synthesis and Characterization

Silica nanoparticles were prepared by hydrolysis of tetraethyl orthosilicate (TEOS, Sigma-Aldrich, Merck KGaA, Darmstadt, Germany)) in ethanol (Carl Roth, Karlsruhe, Germany) in the presence of ammonia (Carl Roth, Karlsruhe, Germany). The protocol was adapted from [44]. Firstly, solution containing ethanol (494 mL), ammonia (72 mL) and deionized water (198 mL) was stirred for 5 min to ensure complete mixing. Second, TEOS (36 mL) was added to the above solution and the reaction was proceeded at ambient temperature for 1.5 h. Thereafter, the colloidal solution was separated by centrifugation, and the silica particles were washed with absolute ethanol for three times to remove the residues, followed by drying in an oven at 70 °C for 12 h.

## 3. Results

Here we present the results for two proof-of-principles for the development of single- and multi- bacterial motile and sensing biohybrids derived from *B. subtilis*. In the first part, we show that single *B. subtilis* cells and chains of cells adhered to the cargoes and provided the required thrust to transport them. In the second part, we demonstrate that genetically engineering signal transduction systems in *B. subtilis* allowed us to equip these biohybrids with the necessary molecular tools to communicate the presence of compounds of interest in the surrounding media while actively carrying the cargo.

### 3.1. Binding and Transport

#### 3.1.1. *Bacillus subtilis* as a Carrier for Biohybrid Microswimmers

**Figure 3:**
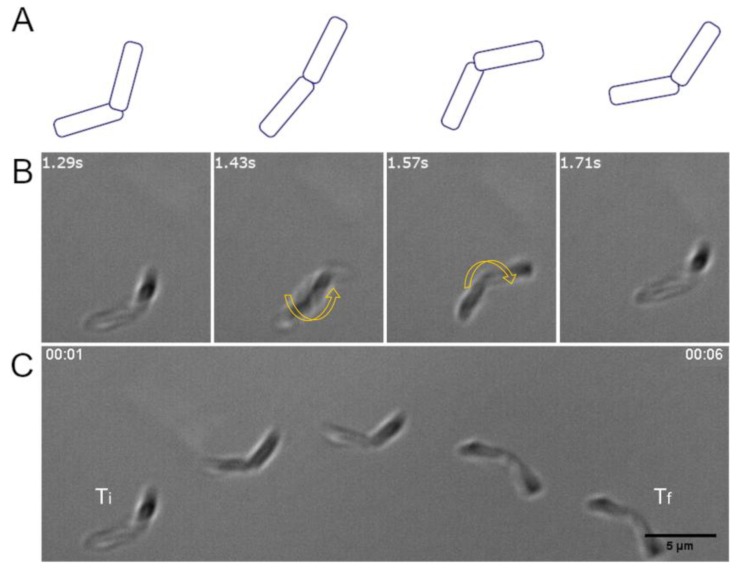
*B. subtilis* swimmer constituted by two chained cells swimming by bending up and down. (**A**), schematics and (**B**), corresponding microscopy images illustrating the up and down bending movement (indicated by the two yellow arrows) of two *B. subtilis* chained cells swimming. (**C**), time lapse microscopy images of the resulting swimming pattern. Ti, initial time; Tf, final time. The images in (**B**) correspond to the first frames of the time lapse presented in (**C**). Strain W168. Scale bar 5 µm for all images. White numbers indicate time in seconds.

It has been previously described that swarming bacterial cells are longer and more flagellated than swimming cells [45]. Since this differentiation phenotype might be helpful for the qualities of biohybrids, we induced the swarming state of our *B. subtilis* strains and characterized the swimming behavior of cells resuspended from the leading edge of the swarming colonies, prior to investigate their potential as biohybrid carriers. Interestingly, our results showed that in addition to single *B. subtilis* cells swimming in the characteristic run-and-tumble mode, swimmers consisting of bacterial cell pairs displaying up-and-down bending where also very frequent (Figure 3). Even chains of up to four bacteria were found, but they were rare.

**Figure 4:**
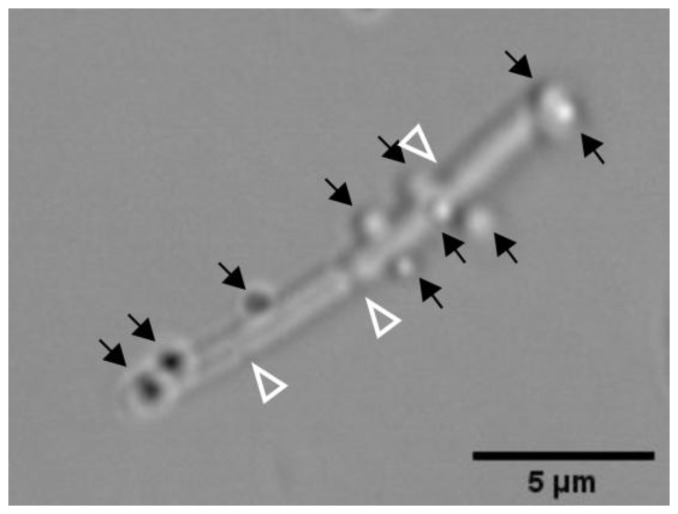
Silica particles attached to two chained *B. subtilis* cells that are already starting to divide again. The bacteria-particle suspensions were incubated at 37 °C (550 rpm) for at least 30 min to promote bacteria-particle interactions prior to the microscopic observation. The abiotic particles are indicated with full black head arrows. Bacterial septa are indicated with empty white head arrows. Phase contrast image of strain TMB3909. Scale bar is 5 µm.

**Figure 5:**
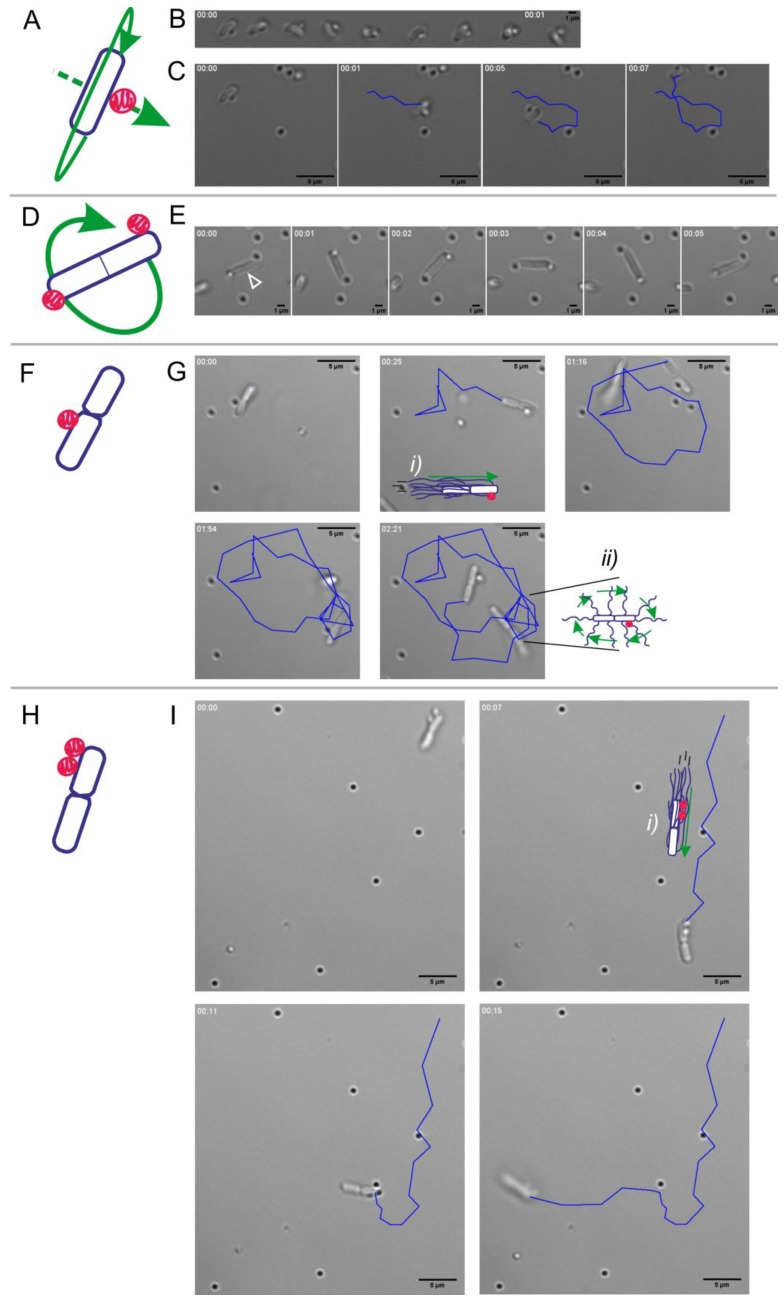
Natural variations in *B. subtilis* W168 biohybrid microswimmers designs. (**A**–**C**), schematics (**A**) and optical microscopy (**B**,**C**) of a biohybrid composed by 1 bacterium:1 cargo rotating over long axis of the cell while moving forward. (**B**), time lapse of the bacterial movement pushing the cargo. (**C**), time lapse of the biohybrid trajectory. The images correspond to Video S2 in the Supplemental Materials. (**D**,**E**), schematics and optical microscopy of a dividing bacteria with two cargoes in opposite sites rotating over long axis (Video S3 in Supplementary Materials). The incipient septum is indicated with a white empty head arrow. (**F**,**G**), schematics and microscopy pictures of a bacterial pair with one cargo attached to the leading cell, performing run (***i***) and tumbling (***ii***) (Video S4 in Supplementary Materials). (**H**,**I**), schematics and microscopy images of a bacterial pair with two cargoes attached to the rear cell, running (***i***) but changing direction without tumbling (Video S5 in Supplementary Materials). Schematics (**A**,**D**,**F**,**H**) for the bacteria-particles biohybrids composition (blue represent de bacterium and pink represents the cargo) and the types of movements (green arrows) are given for clarification; moving trajectories are indicated in blue. Time is indicated in white in the upper left corner. Scale bar in (**B**) and (**E**) images is 1 µm; for all the other microscopy pictures the scale bar is 5 µm.

Next, we tested if *B. subtilis* could act as a biohybrid carrier able to bind and transport a cargo of interest. Towards this end, we incubated our swimmers with silica particles of approximately 800 nm in diameter. The size of the particles was selected to enable the use of optical microscopy to track the motion of the biohybrids. Microscopical observation of the bacteria-particles preparations showed that not only *B. subtilis* naturally adhered to the silica particles (Figure 4) in a reproducible manner, but also that the binding was very stable over time (Supplementary Video S1). Furthermore, *B. subtilis* cells were able to generate the thrust necessary for the transport of more than one cargo particle (Figure 5).

#### 3.1.2. Variations in the Bacteria-to-Object Ratio Resulted in Different Natural Biohybrid Designs

Figure 5 shows phase-contrast microscopy images of different biohybrid microswimmers composed of one or two *B. subtilis* cells with one or two abiotic cargoes (see schematics), surrounded in some cases by free swimming bacteria (Figure 5G) and unloaded particles (either in Brownian motion or deposited at the bottom of the plate). Figure 5C,G,I illustrate the trajectories of the freely swimming bacterial-driven biohybrids to whom no external directional control was applied to steer them.

*B. subtilis* biohybrids varied in the bacteria-to-particle ratio, which resulted in different movement patterns. Figure 5A–C shows a Bacteria Bot (1 bacterium:1 particle) biohybrid pushing the cargo from behind by torquing movement over the long axis of the cell accompanied by forward displacement at a swimming velocity of 3.7 µm s^−1^. The same rotation along the long axis of the cell but without an actual longitudinal displacement was present in dividing bacteria with two particles attached at opposite poles of the cell, that instead remained in place (Figure 5D,E). In that case, the carried cargoes were displaced at a velocity of 2.6 µm s^−1^. Figure 5F,G illustrates a biohybrid constituted by two chained bacteria with a single cargo that performed running and tumbling movements when swimming at 0.8 µm s^−1^ (running and tumbling movements are indicated inside the microscopy pictures as cartoons (*i*) and (*ii*)). However, Figure 5H,I shows two chained bacteria with two cargoes that changed direction after a run (*i*) without being able to perform a full tumbling event, but with a swimming velocity of 4.7 µm s^−1^. It is worthwhile noting that in the cases of pairs of bacteria acting as carriers over longitudinal displacements, the cargoes could be carried both by the leading (Figure 5G) or by the lagging cell (Figure 5I) when considering the direction of the movement. Also, that the swimming velocity of the biohybrids was not correlated with the number of cargoes, nor with the number of bacteria acting as carriers. This suggest that biohybrid performance might be determined by the cellular state of the carrier and/or the conditions of the microenvironment.

### 3.2. Sensing and Responding

Having demonstrated that *B. subtilis* can provide the tracking force to function as a biohybrid carrier, we next exploited the full potential of its genetic toolbox [39,46] to demonstrate that these biohybrids can in fact be equipped with communication abilities. For this proof-of-concept, we selected two whole-cell biosensors derived from the very-well characterized signal transduction pathways of the two-component systems LiaRS and BceRS [28].

#### 3.2.1. *B. subtilis* Whole-Cell Biosensors for Biohybrid Systems: Biochemical Models

Here, we briefly describe the molecular biology required to understand the two genetically engineered signal transduction pathways used for the creation of the whole-cell biosensors applied in this study.

**Figure 6:**
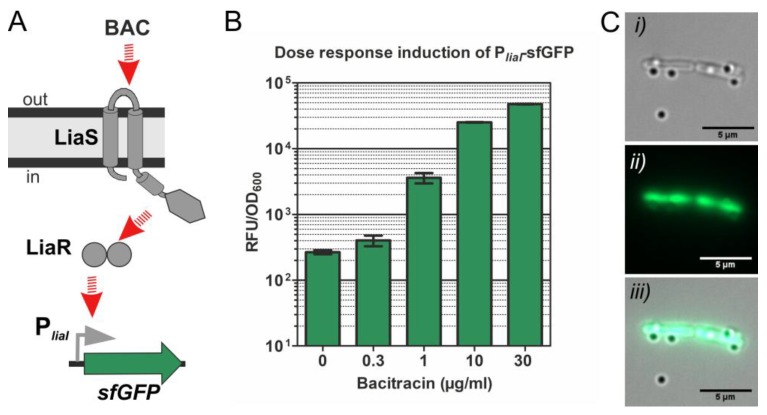
Whole-cell biosensor with dose-response readout. (**A**) Schematics for the whole-cell biosensor P*_liaI_*-*sfGFP* derived from the two-component system LiaRS. The gene coding for sfGFP is cloned downstream of the *liaI* promoter. BAC, stands for the inducer bacitracin. Red arrows indicate activation. See main text for details about the complete signal transduction process. (**B**) Dose-response induction of P*_liaI_*-*sfGFP* (strain TMB3909) by increasing concentrations of the antimicrobial peptide bacitracin. The output is expressed as relative fluorescence units (RFU) divided by the amount of cells as determined by the optical density of the culture at 600 nm (OD_600_). (**C**). Silica particles attached to the whole-cell biosensor P*_liaI_*-*sfGFP* (strain TMB3909) induced by bacitracin 30 µg mL^−1^; (***i***) phase contrast image, (***ii***) green fluorescence, (***iii***) merged images. Scale bar is 5 µm.

**Figure 7:**
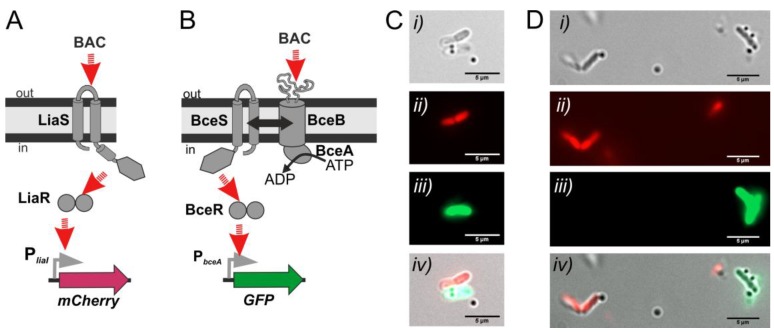
*B. subtilis* biosensors attached to abiotic particles and communicating the presence of bacitracin by red and green fluorescence readout. Schematics for the whole-cell biosensor P*_liaI_*-*mCherry* (**A**) and whole-cell biosensor P*_bceA_-GFP* (**B**) derived from the two-component systems LiaRS and BceRSAB, respectively. The genes coding for mCherry and GFP are cloned downstream of the *liaI* and *bceA* promoters. BAC, stands for the inducer bacitracin. Red arrows indicate activation. See main text for details about the complete signal transduction process. (**C**,**D**), microscopy examples of two genetically different populations of biohybrids, P*_liaI_*-*mCherry* (strain TMB3916) and P*_bceA_-GFP* (strain TMB2174) within the same sample communicating the presence of bacitracin (18 µg mL^−1^) by red and green fluorescence readout; (***i***) phase contrast image, (***ii***) red fluorescence, (***iii***) green fluorescence, (***iv***) merged images. Scale bar is 5 µm.

The Lia system comprises the TCS LiaRS and its target genes *liaIH* [36,47]. Upon induction, the HK LiaS activates the RR LiaR, which then drives the expression of P*_liaI_*, the only target promoter of the system [47,48]. Comprehensive studies on the dynamics of the system and the well-established genetic toolboxes based on the Lia system highlight its potential and usefulness for the creation of whole-cell biosensors [28]. For the development of our sensing-biohybrids we used two different strains engineered from the LiaRS TCS from *B. subtilis*: strains P*_liaI_-sfGFP* (TMB3909) and P*_liaI_-mCherry* (TMB3916), containing the *liaI* target promoter of LiaR response regulator upstream of *sfGFP* (Figure 6A) and *mCherry* (Figure 7A) coding genes, respectively [39].

The second signal transduction pathway applied in this study is the four-protein module BceRSAB. The TCS BceRS consists of the histidine kinase BceS and the response regulator BceR, which together with the associated ABC transporter BceAB constitute an antimicrobial peptide sensing and detoxification unit [37,49]. Here, we used the strain P*_bceA_-GFP* (strain TMB2174), containing the target promoter of the RR BceR upstream of the GFP reporter gene (Figure 7B).

Both systems, BceRS and LiaRS, are activated by a plethora of antimicrobial peptides. For example, inducing substrates of BceRS are bacitracin, plectasin, actagardine and mersacidin, while LiaRS is induced e.g., by bacitracin, enduracidin and ramoplanin [29]. The target promoters of both systems show a very low basal activity in the absence of stimulus, and their activation is dose sensitive. Figure 6B shows the dose-dependent induction of the whole-cell biosensor P*_liaI_-sfGFP* in response to increasing concentrations of the antibiotic bacitracin.

#### 3.2.2. Genetically Engineered Bacterial-Hybrid Biosensor Microswimmers

Finally, we aimed to develop bacterial biohybrids with sensing and communication properties. For that, we incubated *B. subtilis* whole-cell biosensor swarming cells with the abiotic particles in the presence of inducing stimulus. Phase-contrast and fluorescence microscopy was carried out after the incubation periods.

**Figure 8:**
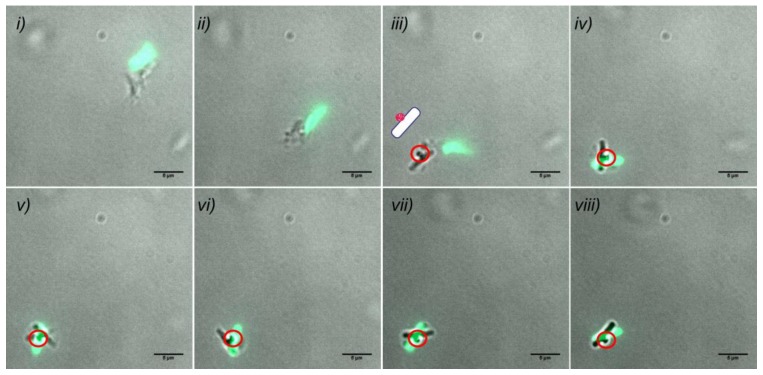
*B. subtilis* hybrid biosensor microswimmer. Merged images of green fluorescence and phase contrast time-lapse microscopy (8-frame time series, indicated ***i*** to ***viii***). The images illustrate the displacement of the freely swimming biosensor microswimmer P*_bceA_-GFP* (strain TMB2174) indicating the presence of bacitracin (18 µg mL−^1^) in the surrounding media by the expression of green fluorescence, while carrying one inert cargo attached to its surface (the schematics of the Bacteria Bot composition is included in image (***iii***); the cargo is circled in red from (***iii***) to (***viii***)). Note that due to technical limitations there is a delay between the phase contrast movement images and the fluorescence readout. The complete time-lapse series can be seen in Video S6 in the Supplementary Materials. Scale bar is 5 µm.

Figure 6C shows the whole-cell biosensor P*_liaI_*-*sfGFP* with six cargoes attached to its surface; the carrier bacteria are fluorescently labelled due to the activation of the LiaRS TCS signal transduction pathway by bacitracin, resulting in the expression of green fluorescence protein (Figure 6A). Furthermore, the combination of different biosensors within the same preparation allowed us to visualize two genetically different populations of biohybrid biosensors with different fluorescence readouts due to the activation of two different signal transduction pathways. Figure 7C,D show examples of the biohybrid-biosensors P*_liaI_*-*mCherry* and P*_bceA_-GFP*, the former one showing red fluorescence due to the activation of LiaRS TCS resulting in the expression of mCherry protein (Figure 7A), while the latter one exhibits green fluorescence due to the activation of the BceRS module leading to the expression of green fluorescence protein (Figure 7B). Additionally, Figure 8 shows the swimming trajectory (with a running phase at 1.6 µm s^−1^ (Figure 8*i*–*iii*) and a tumbling event (Figure 8*iv*–*viii*)) of biohybrid P*_bceA_-GFP* carrying one cargo attached to its surface and showing green fluorescence (schematics for the Bacteria Bot composition is included in Figure 8*iii*) after activation of the BceRS signal transduction pathway by bacitracin (Figure 7B).

It is worthwhile noting that sensing and movement are two different and independent processes occurring simultaneously in the cell, as shown in Figure 8. Further, our results demonstrate that sensing abilities are not affected by binding to the particles as depicted in Figure 6C, Figure 7C,D, and Figure 8 where fluorescence readout is achieved even in the presence of more than one cargo attached to the bacteria. Altogether, our results show that the genetically engineered signal transduction pathways of *B. subtilis* allowed us to create hybrid biosensing microswimmers able to couple the binding and transport of abiotic particles with sensing and communicating the presence of molecules of interest in the surrounding media, in a detectable and measurable way by means of fluorescence expression.

## 4. Discussion

The ability to perceive and react to environmental cues in an autonomous way is one desired property of microswimmers. In this study, we have demonstrated that the QPS/GRAS organism *B. subtilis* [50] can actually be used as a vehicle for cargo transport, giving rise to different motion patterns depending on bacteria (single or chained) and cargo multiplicity. Second, we have shown that this vehicle can be engineered to perceive and respond to certain aspects of its environment.

As stated in the introduction, three critical steps challenge the development of bacterial biohybrid microswimmers—the attachment, the delivery, and the release of the cargo (Figure 1)—and a desired property is autonomous sensing of and responding to environmental clues. Since the focus of this study was on the biosensing capabilities of the *B. subtilis* cells, a simple cargo particle was used. Unmodified silica particles with a diameter of 800 nm showed efficient binding to the *B. subtilis* W168 strain (Figure 4). This was initially surprising, since the surface charge of both, the cells and the particles is negative and therefore not in favor of binding. However, *B. subtilis* was shown to form silicate precipitates at its surface [51,52]. These silicates are proposed to bind to the cell surface by covalent bonds, such as esters and acid anhydrides. In our working model, binding of *B. subtilis* W168 to unmodified silicate particles could be due to covalent bonds, rather than electrostatics; being beyond the scope of this work, the actual nature of the binding reported here and the concomitant strategies for cargo release will be the focus of further investigations. Nonetheless, the attachment of the silica particles to *B. subtilis* surface was stable over time and the bacteria were able to provide the required thrust to carry one or several cargoes showing different patterns of displacement (Figure 5). Bacterial Microsystems containing more than one bacterium attached to and carrying a single cargo have been described previously, such as the bacterial carpets [7,53] or bacterial-attached microbeads [54,55]. Interestingly, most of our *B. subtilis* biohybrids were at the same time Bacterial Microsystems, naturally composed by two chained bacteria, and Multi Bots, carrying more than one cargo (Figure 5).

TCSs are promising molecular tools to develop bacterial biohybrid microswimmers able to sense and respond to a plethora of specific environmental stimuli. The abundance of TCSs encoded in the genome of a bacterium might vary from very few to over 100 (e.g., *B. subtilis* encodes 36 histidine kinases [56]), and it usually reflects the complexity of the environment inhabited (a higher amount of TCSs is required to survive in more demanding, that is, rapidly changing environments) [57]. Whole-cell biosensors like the ones applied here, make profit of the fact that bacterial sensor kinases are able to recognize a variety of signaling molecules ranging from metabolites to antibiotics, light, temperature, oxygen, toxic substances, etc. [26,58]. Antibiotic sensing was used as a proof-of-concept in the present work to illustrate the potential of TCSs in the field of microswimmers. Opening the scope for potential applications, TCS-based biosensors able to detect contaminants in environmental samples [59], for example, would be very useful to develop biohybrids for cleaning polluted waters [60].

The genetic tools used in this work adhere to the genetic BioBrick standard, thereby allowing to rapidly generate, fuse and exchange biological parts to fulfill certain functions [61]. Furthermore, applying standardized genetic parts allows their use in a broad variety of different chassis, beyond the use of *B. subtilis*, and opens up the creation of biohybrids with novel functionalities [62]. The modularity of this concept is of particular importance, since it allows to rapidly implement the engineering driven principles such as abstraction and standardization [63]. To date, a collection of more than 12,000 of such individual BioBricks are stored and available from the Parts registry (available at parts.igem.org). Each submitted part contains background information on its generation, design and experimental characterization [39]. This allows convenient rearrangement of the genetic parts of the biohybrids according to the needs of the individual application. For example, exchanging the fluorescence-based reporters described in this work by a luminescence readout. Both fluorescence and luminescence could be useful beyond the mere function as readout of a sensing process. The specific wavelengths emitted by the reporter proteins could be exploited for photochemical follow up reactions. Cargo could be bound to the cells by photo-cleavable linkers [64]. After sensing the stimulus and producing the reporter protein, the emitted light would cleave the linker and release the cargo. Alternatively, the signal transduction pathway could be engineered so that the activation of the TCS leads to the expression of a protease of interest able to degrade a specific peptide linker. Upon a certain stimulus, the activation of the system would lead to protease expression, concomitant proteolysis of the linker and final cargo release.

## 5. Conclusions

In the present work, we have demonstrated that the QPS/GRAS-approved Gram-positive bacterium *B. subtilis* is a genetically versatile and robust microorganism for the development of single- and multi-bacterial biohybrid microswimmers. Genetic engineering tools were applied to tune the natural TCS signal transduction pathways to equip our *B. subtilis* biohybrids with sensing and read-out abilities, increasing the tasks they can perform, and expanding the scope for future developments of bacterial-hybrid biosensor microswimmers.

## Figures and Tables

**Table 1:** Bacterial strains used in this study.

*B. subtilis* Strain	Description ^a^	Source or Reference
W168	Wild type 168 (trpC2)	Mascher laboratory stock
P*_bceA_*-GFP	W168 pJR3802 (P*_bceA_*-gfp), *mls*	TMB2174 ^b^ [38]
P*_liaI_*-sfGFP	W168 *amyE:cat* P*_liaI_*-sfGFP SP	TMB3909 ^b^ [39]
P*_liaI_*-mCherry	W168 *amyE:cat* P*_liaI_*-mCherry BSU	TMB3916 ^b^ [39]

^a^*mls*, macrolide-lincosamide-streptogramine resistance; *cat*, chloramphenicol resistance. ^b^ Reference number from the Mascher laboratory strain collection.

## Supplementary Materials

The following are available online at https://www.mdpi.com/1424-8220/20/1/180/s1, Video S1: Bsubtilis W168 bacteria Bot_stable binding to silica particle. Video S2_Sun et al. Figure 5C_1 bacterium-1 cargo. Video S3_Sun et al. Figure 5E_2 bacteria-2 cargoes-rotation. Video S4_Sun et al. Figure 5G_2 bacteria−1 cargo. Video S5_Sun et al. Figure 5I_2 bacteria−2 cargoes. Video S6_Sun et al. Figure 8_sensing and swimming_composite.
